# Regulation of cell division and expansion by sugar and auxin signaling

**DOI:** 10.3389/fpls.2013.00163

**Published:** 2013-05-30

**Authors:** Lu Wang, Yong-Ling Ruan

**Affiliations:** ^1^Department of Biology, School of Environmental and Life Sciences, The University of NewcastleCallaghan, NSW, Australia; ^2^Australia-China Research Centre for Crop Improvement, The University of NewcastleCallaghan, NSW, Australia

**Keywords:** sugar signaling, auxin signaling, cell division, cell expansion, seed development

## Abstract

Plant growth and development are modulated by concerted actions of a variety of signaling molecules. In recent years, evidence has emerged on the roles of sugar and auxin signals network in diverse aspects of plant growth and development. Here, based on recent progress of genetic analyses and gene expression profiling studies, we summarize the functional similarities, diversities, and their interactions of sugar and auxin signals in regulating two major processes of plant development: cell division and cell expansion. We focus on roles of sugar and auxin signaling in both vegetative and reproductive tissues including developing seed.

## INTRODUCTION

Plant growth and development results from a combination of three processes at the cellular level: cell division, cell expansion, and cell differentiation. Cell division or mitosis involves the duplication and separation of complete sets of genetic materials. This genetic information is then selectively transcribed and translated to determine the final shape of cells through cell expansion and differentiation. Cell division and expansion determines cell number and cell size in a mature organ, hence its yield. Over the last decade, genetic analyses and genome-wide gene expression profiling studies have significantly advanced our understanding of the signaling pathways regulating cell proliferation and expansion. In this context, the phytohormone auxin plays prominent roles in regulating both cell proliferation and cell expansion (reviewed in Perrot-Rechenmann, 2010). Rapid advances in the area have helped shed light on the molecular mechanisms regulating auxin homeostasis, transport, and signaling.

Sugars, in addition to their fundamental roles as carbon and energy sources, also act as signaling molecules to regulate gene expression (e.g., Rolland et al., 2002; Hartig and Beck, 2006). The disaccharide sucrose (Suc) is transported through phloem from photosynthetic leaves (source) to sink organs such as root, meristem, flower, developing fruit, and seed. In general, lowered Suc levels stimulate source activities, including photosynthesis, nutrient mobilization, and export. In contrast, higher Suc levels is believed to inhibit photosynthesis in source leaves, but stimulate growth and storage in sink tissues. Phloem unloading of Suc in companion cell and sieve element (CC/SE) complex and its post-phloem transport to recipient sink cells may occur either apoplasmically into cell wall matrix or symplasmically through plasmodesmata. Prior to its use for metabolism and biosynthesis, Suc must be degraded either by invertase (Inv) into glucose (Glc) and fructose (Fru), or by Suc synthase (Sus) into UDP-glucose (UDPG) and Fru. Based on the subcellular localization, Inv is usually divided as cell wall Inv (cw-Inv), vacuolar Inv (vac-Inv), and cytosolic Inv (cyto-Inv; but it may also be expressed in mitochondria, plastids, and nuclei); while Sus may exist as soluble protein in cytoplasm (cyto-Sus) or insoluble isoform bound to the plasma membrane (PM-Sus; Cai et al., 2011) and other intracellular organelles (Ruan, 2012). These sucrolytic enzyme activities are not only critical for primary and secondary metabolism by supplying essential energy and building blocks for plant growth, but also play direct roles in signaling (Ruan, 2012), since Suc and its cleavage hexose are also signaling molecules regulating gene expression (reviewed by Gibson, 2005; Rolland et al., 2006; Eveland and Jackson, 2012). So far, the best studied plant sugar signaling pathway is Glc signaling, mediated by its sensor hexokinase (HXK), which sequentially regulates plant gene expression at transcriptional, translational, and post-translational levels (e.g., Sheen, 1990; Ho et al., 2001; Yanasigawa et al., 2003). In the past few years, a large number of sugar-responsive loci have been identified by genetic approaches, and different sugar-based signaling pathways are deciphered in specific developmental process (reviewed in Smeekens et al., 2010). Mounting evidence also suggests crosstalks among sugar and various hormone signals, e.g., abscisic acid, ethylene, and cytokinins (Gazzarrini and McCourt, 2001; Hartig and Beck, 2006). Among these, close interactions between sugar and auxin signaling play major roles in various aspects of plant development (Eveland and Jackson, 2012).

This review aims to evaluate recent progress on the regulatory roles of sugar and auxin signaling and their interactions in cell division and expansion from a developmental perspective in both vegetative and reproductive organs.

## AUXIN AND SUGAR SIGNALING IN CELL PROLIFERATION

**FIGURE 1:**
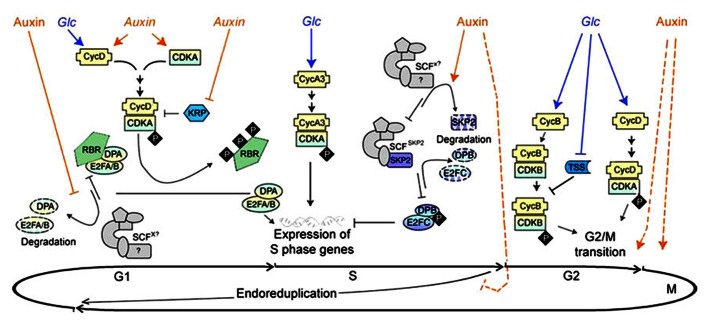
**Glucose and auxin signal in cell cycle regulation (modified from Perrot-Rechenmann, 2010, **Figure 2**).** The cell cycle is divided into four phases: DNA replication (S), mitosis (M), and two gap phases (G1 and G2, between M/S and S/M, respectively). Some plant cells may skip the M phase under certain developmental processes, resulting “endoreduplication.” Cell cycle starts in G1. During this phase, Glc and auxin signals could induce the expression of *CycD*, while auxin is also able to increase *CDKA* transcription. The CycD/CDKA complex is activated by phosphorylation but can still be blocked by CDK inhibitor KRPs. Auxin was reported to reduce the expression of some *KRPs*. The active CycD/CDKA complex provokes phosphorylation of the transcriptional repressor RBR, and release the transcription regulator E2FA/B and DPA complex. By post-transcriptional regulation, auxin stabilizes the E2FA/B and DPA complex, which promote the expression of genes essential for the beginning of the S phase. The expression of CycA3 could be up-regulated by Glc signal, which is required to drive the cells from G1 to S phase. Auxin was shown to increase the degradation of the F-box SKP2 later in S phase, which indirectly stabilizes E2FC/DPB complex, and represses the S phase genes expression. As cell cycle processes into G2 phase, Glc signal was found to initiate the G2/M transition by repressing *TSS* transcription, and activating the expression of key cell cycle genes, such as *CycB* and *CycD*. Auxin signal is required for the initiation and completion of mitosis, probably though an unknown pathway independent or in parallel to Glc. Auxin is also likely to emit a negative signal to prevent cell from going into endoreduplication hence sustaining cell divisions. Glc and Auxin in italic indicate regulation at transcription level and those in non-italic suggest regulation at protein level.

The eukaryotic cell cycle consists of DNA synthesis (S phase) and mitosis (M phase), separated by two gap phases G1 and G2 (**Figure 1**). Mitogenic signals are required for completion of cell cycle, in particular during the transitions from G1 to S and G2 to M phases for proper progression of the cycle; otherwise the cell cycle will be arrested. Some plant cells may skip the M phase under certain developmental processes resulting in endoreduplication and an increase in the degree of ploidy (**Figure 1**; Joubés and Chevalier, 2000). A typical example of cell cycle without mitosis occurs in the syncytial endosperm early in seed development.

As a class of essential plant hormones, auxin has been demonstrated to play a leading role in regulating cell proliferation, especially in the preparation of replication (G1 to S phases). A set of *in vitro* cell culture studies have provided insights into the molecular mechanisms by which auxin regulates cell cycle (**Figure 1**; reviewed in Perrot-Rechenmann, 2010). During G1 phase, auxin was shown to induce the expression of cyclin D gene *cycD3;1* and cyclin-dependent kinase gene *CDKA;1*, and to play important roles in CDKA/CYCD complex assembling. Meanwhile, *KRP1* and *KRP2* transcripts, encoding two of the CDK inhibitors, were reported to be down-regulated after auxin treatment, thereby preserving the phosphorylated CDKA/CYCD complex. Activated CDKA/CYCD complex could provoke phosphorylation of the transcriptional repressor retinoblastoma-related (RBR) protein, and release its target Adenovirus E2 promoter-binding factor A/B (E2FA/B) and dimerization partener A (DPA) complex. Through this post-transcriptional regulation, auxin stabilizes the E2FA/B and DPA complex, which promotes the expression of genes essential for initiating the S phase. Later in the S phase, auxin stimulates the degradation of the F-box SKP2A (S phase kinase-associated protein 2A) by E3 ubiquitin ligase complex SCF (Skp, Cullin, F-box containing complex), indirectly stabilizing the E2 promoter-binding factor C (E2FC) and dimerization parterner B (DPB) complex. The latter represses the expression of S phase genes. Although most data suggest auxin acts as a permissive signal for achieving competence to enter DNA synthesis (G1/S transition), it is also required in the later G2/M transition to complete the mitosis process (**Figure 1**; Urano et al., 2012). However, it is difficult to dissect the effect of auxin on later phases from that at the initial step of the cell cycle. Consistent with its role in expedition of cell cycle process, auxin was also found to promote cell division and delay endoreduplication in developing seeds of legume species *Medicago truncatula* (**Figure 1**; Atif et al., 2012).

Comparing to auxin, our current knowledge about the molecular mechanism of sugar-mediated regulation of cell division is largely derived from studies on cultured suspension cells and mutant seedlings subjected to various sugar treatments. A close correlation was observed between the supply of Glc and the expressions of cyclins, e.g., *cycD2;1*, *D3;2*, *A3;2*, and *B1;2* (**Figure 1**; Riou-Khamlichi et al., 2000; Hartig and Beck, 2006). The D-type cyclins are often mentioned as sensors of external conditions, and associate with cyclin-dependent kinase (e.g., CDKA) to regulate cell cycles (Nieuwland et al., 2007). Meanwhile, A3 and B1 cyclins are required to drive G1/S and G2/M transitions, respectively (Menges et al., 2005). These observations suggest that Glc signaling regulates cell cycle throughout the whole cell cycle process. Noteworthy is that the regulatory effect of Glc on the rate of cell division primarily results from signaling rather than nutrient availability and energy status, as cell proliferating activity positively correlated with endogenous hexose levels, but not their uptake rate (Hartig and Beck, 2006). A recent study in *Arabidopsis* meristematic tissues has shown that Glc signal initiates the G2/M transition by repressing transcription of the negative regulator *TPR-DOMAIN SUPPRESSOR OF STIMPY* (*TSS*), thereby activating the expression of key cell cycle components required for G2/M transition, such as *CYCB1;1* and *CDKB1;1* (**Figure 1**; Skylar et al., 2011). Noteworthy is that Glc feeding is insufficient to trigger mitosis, and auxin is also required for the completion of this process, indicating distinct but coordinated roles of sugar and auxin in G2/M regulation (Skylar et al., 2011).

Other than hexose, downstream components of Suc/Glc signaling factors may also be involved in cell cycle regulation. Trehalose-6-phosphate (T6P) is a newly identified signal molecule which is synthesized from G6P and UDPG by T6P synthase (TPS; Paul et al., 2008). AtTPS1, being the only functional *Arabidopsis* TPS enzyme catalyzes T6P synthesis reaction, was observed interacting with CDKA1 and the kinesin KCA1, while its loss of function mutant *tps1* shown embryo lethal (reviewed in Smeekens et al., 2010). The exact role of T6P in cell cycle remains unclear. However, a downstream target of T6P, Suc non-fermenting1 (Snf1)-related protein kinase (SnRK1), is considered to be a sensor negatively regulating plant growth through crosstalk with cell cycle signaling factors as indicated by studies of its homolog Snf1 in yeast (Francis and Halford, 2006). An inhibition of the catalytic activity of SnRK1 by T6P was revealed both *in vitro* and *in vivo* (see O’Hara et al., 2013). A link between T6P/SnRK1 regulatory system and auxin signaling has also been revealed (discussed later, reviewed in O’Hara et al., 2013), implying a potential molecular mechanism of T6P and SnRK1 signaling in regulating cell cycle. However, SnRK1 signal is currently associated with the response to starvation or low energy status during plant development, as its activity leads to down-regulation of carbon-consuming processes but enhancement of photosynthetic processes, thereby increasing carbon availability (Halford and Hey, 2009; O’Hara et al., 2013). It remains to be determined whether SnRK1 signal plays a role in cells division. Similarly, the perception of cellular energy and nutrient levels typically leads to the activation of the growth promoting target of rapamycin (TOR) protein kinase signaling pathway, which then adjusts cell growth and proliferation accordingly. It has been reported that TOR promotes *Drosophila* female germline stem cells proliferation during G2 phase (LaFever et al., 2010). A single copy of TOR has been found in *Arabidopsis*, the loss of which results in embryonic lethality (Menand et al., 2002). Whether the TOR complex functions in regulating plant cell division remains to be investigated.

## ROLES OF AUXIN AND SUGAR IN CELL EXPANSION

Cell expansion is another important cellular process for plant growth, which is a net result of internal turgor pressure and irreversible cell wall extension. By accumulating sugars, irons and other osmotically active solutes, plant cell generates a lower osmotic potential to attract water flux into the cell, thereby generating a turgor pressure to drive cell expansion. This process also requires the cell wall to be irreversibly stretched through a wall loosening process, followed by deposition of new wall material. The extent and direction of cell expansion is modulated by many factors including cytoskeletons (Li et al., 2001; Pollard and Cooper, 2009).

**FIGURE 2:**
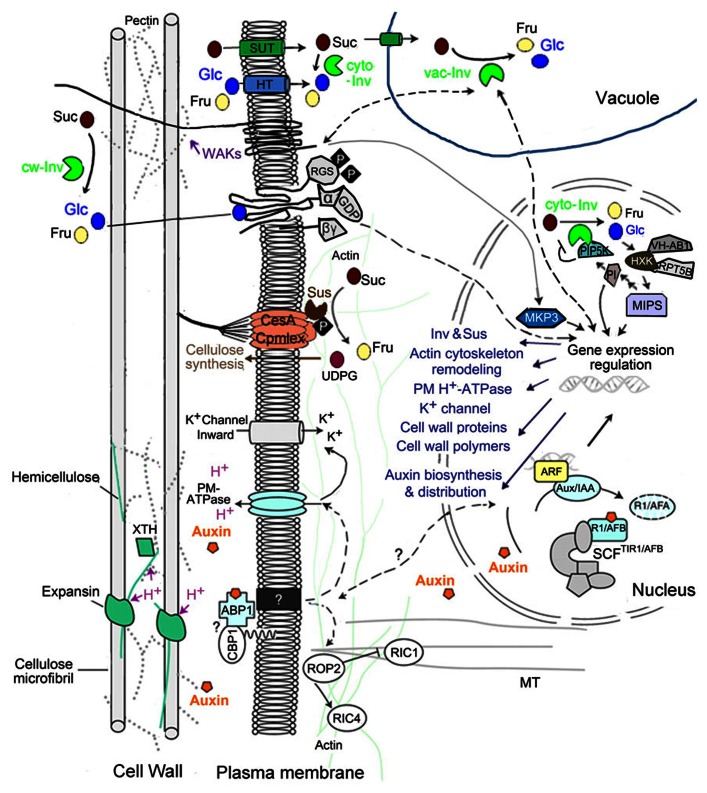
**Sugar and auxin signal in regulating sink cell expansion (modified from Perrot-Rechenmann, 2010, Figure 3).** Unloaded Suc in sink tissues may enter into recipient cells either apoplasmically through cell wall matrix or symplasmically via plasmodesmatal. In the former case, sucrose could be taken up by sucrose transporter on plasma membrane, or be hydrolyzed by cw-Inv into Glc and Fru, and then be transported into cells by hexose transporter (HT). Apoplasmic Glc could be recognized by RGS1, which transmits extracellular sugar signal into the cell through G-proteins. In cytoplasm, Suc may be hydrolyzed by cyto-Inv or degraded by Sus. In the present of high Suc level, Sus intends to bind actin filaments and form a multi-protein complex bound to plasma membrane, which may facilitate cell expansion by providing UDPG for cellulose/callose biosynthesis. Cytoplasmic Suc could also be transported into nucleus, vacuole, plastid or mitochondrion. In vacuole, Suc could be hydrolyzed by vac-Inv, thus doubling the osmotic contribution of Suc, which has the potential to positively impact on cell turgor. Moreover, vac-Inv could also promote cell expansion via sugar signaling involving WAKs, which subsequently activates MPK3 in nucleus, and induces downstream gene expression for cell wall biosynthesis. Suc hydrolysis in vacuole could also regulates nuclear gene transcription, involving in auxin biosynthesis, distribution and signaling. An *Arabidopsis* cyto-Inv isoform was found in nucleus, where it interacts with and negatively regulated by a phosphatidylinositol monophosphate 5-kinase (AtPIP5K9). Hexoses generated by cyto-Inv could be sensed by a nuclear-localized HXK, producing a Glc signaling complex core combining VHA-B1 and RPT5B, which is sequentially integrated into a signal/metabolites loop modulating cell expansion. Auxin is perceived by the auxin receptor ABP1, which interacts with unknown membrane-associated proteins at the plasma membrane [such as glycosylphosphatidylinositol (GPI)-anchored protein C-terminal peptide-binding protein 1. (CBP1)]. This activates the proton pump ATPase, acidifying extracellular space for optimal function of expansins and XTH and activating K^+^ inward rectifying channels, essential for water uptake to sustain cell expansion. Auxin could also enhance these effects by promoting the transcription of these genes. Moreover, auxin is likely to act on actin microfilaments and microtubules via the modulation of ROP GTPases, thereby affecting vesicle delivery to plasma membrane and cell wall matrix.

Auxin was shown to induce rapid cell elongation in stem, coleoptiles, or hypocotyls segments within minutes after auxin treatment (Rayle and Cleland, 1992). Current model of auxin-regulated cell expansion is based on an acid growth theory (**Figure 2**; reviewed in Mockaitis and Estelle, 2008; Perrot-Rechenmann, 2010). An extracellular localized auxin is perceived by the auxin receptor, auxin binding protein 1 (ABP1). An interaction between the ABP1 and some unknown membrane-associated proteins may activate the plasma membrane (PM)-H^+^-ATPase, pumping proton into the extracellular space. This lowers the pH in cell wall matrix, activating cell wall loosening proteins such as expansins and xyloglucan endotransglycosylase/hydrolases (XTH), and consequently making the cell wall relaxed for expansion. The PM-H^+^-ATPase activity also promotes hyperpolarization of membrane potential which activates voltage-dependent potassium inward channels, thereby contributing to the osmotically driven water uptake for expansion.

In parallel to its role in activating proteins essential for cell expansion at the post-translational level, auxin signal in the nuclei also enhances the transcription of these genes including those encoding PM-ATPase, K^+^ channels, expansins, and cell wall remodeling enzymes. Furthermore, auxin promotes exocytosis of vesicles containing new cell wall material (**Figure 2**; reviewed in Perrot-Rechenmann, 2010). In addition, auxin may affect F-actin through ABP1 and its downstream Rho GTPases and their effectors CRIB MOTIF-CONTAINING proteins (RICs), which consequently modulates asymmetric cell expansion (**Figure 2**; Yang and Fu, 2007; Xu et al., 2010).

Different from auxin which regulates cell growth solely through signaling, sugars modulate cell expansion both as important signals and metabolites. The latter serves as osmotically active solutes and substrates for biosynthesis of diverse products including cell wall material required for cell expansion.

 By hydrolyzing Suc into Glc and Fru, vac-Inv doubles the osmotic contribution of Suc in vacuole, and thus has the potential to positively impact cell turgor in cells accumulating hexoses to high levels, such as elongating cotton fiber (**Figure 2**; Wang et al., 2010). Consistently, high vac-Inv expression or activity has been observed in a range of expanding tissues, for example, maize ovaries (Andersen et al., 2002), grape berry (Davies and Robinson, 1996), carrot taproot (Tang et al., 1999), and a reduction of maize ovary expansion was associated with the decrease of a vac-Inv gene *Ivr2* expression under drought (Andersen et al., 2002).

In agreement with the idea that sugar could also act as signal for cell expansion, vac-Inv has been shown to promote cell expansion in *Arabidopsis* root through an osmotic-independent pathway (Wang et al., 2010). One possible explanation is that vac-Inv may crosstalk with wall-associated kinases (WAKs) through sugar signaling to regulate cell wall extensibility (**Figure 2**). Here, as receptor-like proteins, WAKs are bound to pectin in cell walls, and their activity is required for cell expansion (Anderson et al., 2001; Lally et al., 2001). The observations that vac-Inv activity and *AtvacINV2* transcription were dramatically reduced in *Arabidopsis* *wak2-1* mutant (Kohorn et al., 2006), and the deletion of *AtvacINV2* (*vin*, Salk_100813) down-regulated the expression of *AtWAK2* (Wang and Ruan, unpublished data), strongly suggest an interplay between vacuole sugar homeostasis and extracellular matrix signaling during cell expansion. Microarray analysis revealed a WAK2-dependent pectin activation of many genes involved in cell wall biosynthesis, which is likely achieved via a downstream mitogen-activated protein kinase AtMAPK3 (**Figure 2**; Kohorn et al., 2009; Kohorn and Kohorn, 2012). This, together with our finding on repression of WAK expression in *vin* mutant also implies that a sugar signal derived from vacuole could play roles in transcription of genes required for cell expansion. Indeed, Suc hydrolysis in vacuole was able to evoke a sugar signal effect on numerous gene expressions, involving auxin biosynthesis, distribution, and auxin signal sensing (Mishra et al., 2009; discussed below). Suggested by the finding that vacuolar H^+^-ATPase B1 unit (VHA-B1) interacts with the nuclear-localized AtHXK1 (Cho et al., 2006), a similar protein complex on tonoplast in transmitting vacuolar sugar signals might be expected. However, it remains to be determined as to what is the exact downstream signal pathway of this vacuole sugar signal, and how vac-Inv interacts with cell wall protein WAKs to regulate cell expansion.

Other than vac-Inv, cyto-Inv was also described to play a role in regulating root cell elongation in *Arabidopsis* and rice (Lou et al., 2007; Jia et al., 2008). Interestingly, the *Arabidopsis* cyto-Inv isoform AtCIN1 (At1g35580) was observed in nuclei, where it interacts with a phosphatidylinositol monophosphate 5-kinase (AtPIP5K9), and is negatively regulated by AtPIP5K9 (**Figure 2**; Lou et al., 2007). AtPIP5K is a key enzyme in phosphatidylinositol (PI) signaling pathway, and may directly or indirectly regulate cytoskeleton dynamics via myo-inositol (Lou et al., 2007). Meanwhile, the hexoses derived from nuclear Suc degradation catalyzed by AtCIN1, may be sensed by nuclear-localized AtHXK1 (Cho et al., 2006), and sequentially modulates transcriptions of specific target genes (**Figure 2**). However, the biological functions of the nuclear-localized AtCIN1 and AtHXK1 protein complex are still unclear.

In contrast to Inv, the second Suc-degrading enzyme, Sus, contributes to cell expansion primarily through providing one of the cleavage reaction products, UDPG for cell wall biosynthesis. For example, Sus is highly expressed in cotton cellularizing endosperm cells (Ruan et al., 2008) and seed coat transfer cell undergoing wall in growth (Pugh et al., 2010). Recent studies in *Arabidopsis* and tobacco support a model of PM-Sus-mediated cell wall biosynthesis where PM-Sus binds to actin filaments to initiate the formation of a multi-protein complex and to provide UDPG to callose synthase and cellulose synthase, thus facilitating cell expansion via cellulose/callose biosynthesis (**Figure 2**; Fujii et al., 2010; Cai et al., 2011). Interestingly, a conversion from the cytosolic Sus to its membrane-associated form was detected in the presence of high Suc level, indicating a role of Suc homeostasis in cell wall deposition (Cai et al., 2011). Recently, during *Arabidopsis* seed development, hexose signaling was observed to induce the expression of Sus genes via a HXK-independent pathway (Angeles-Núñez and Tiessen, 2012). Together, these results show that sugars may regulate Sus-mediated cell wall biosynthesis through cellular metabolism as well as signaling network.

Research on sugar signaling has been primarily focused on intracellular processes. It remains virtually unknown how cell senses extracellular signals such as sugars to elicit downstream cellular processes. Ruan et al. (2009) proposed that the apoplasmic Glc generated by cw-Inv could be recognized by a membrane protein, RGS1 (regulator of G-protein signaling 1), which transmits extracellular sugar signal into the cell (**Figure 2**). An insight into the molecular basis of sugar and G-protein signaling crosstalk comes from a recent discovery by Urano et al. (2012) in which Glc could be sensed by AtRGS1 that represses the activity of heterotrimeric G-protein complex. This Glc sensing leads to endocytosis of AtRGS1, hence uncoupling the inhibitory effect of AtRGS1 on AtGPA1 (G-protein α subunit) and consequently activating G-protein signaling (Urano et al., 2012).

Other than G-protein, HXK could also be a potential integrator in regulating cell expansion through Glc signaling, probably via modulating cytoskeleton dynamics (Karve et al., 2008; see **Figure 2**). AtHXK1 was shown to affect F-actin dynamics, thereby influencing the formation and the stability of cytoskeleton-bound polysomes, and the complex membrane trafficking involved in expansion (Balasubramanian et al., 2007). In addition, Cho et al. (2006) suggested a nuclear Glc signaling core formed by nuclear-localized AtHXK1 interacts with VHA-B1 and the 19S regulatory particle of proteasome subunit (RPT5B). *Arabidopsis* VHA-Bs are involved in actin cytoskeleton remodeling via binding and stabilizing F-actin *in vitro* (Ma et al., 2012), thereby potentially affecting cell expansion through modulating actin-guided vesicle trafficking for cell wall synthesis.

Together, the above analyses allow us to formulate a model of cell expansion regulated by sugar and auxin signaling network, covering WAKs, G-protein and nuclear PI signaling, and linking transmission of signals from extracellular environment to different subcellular compartments for a range of cellular processes required for cell enlargement (**Figure 2**).

## THE INTERPLAY OF SUGAR AND AUXIN SIGNALING PATHWAYS

As discussed above, sugar and auxin signals play distinctive roles in regulating cell division and expansion. However, the two pathways also interact with each other to regulate plant development and recent studies indeed show that auxin biosynthesis, distribution, and response is regulated directly by sugar signal.

Several biosynthesis pathways of the main auxin, IAA (indole-3-acetic acid) have been postulated in plant. These include the widely distributed IAM (indole-3-acetamide) pathway, the IPA (indole-3-pyruvic acid) pathway, and two possible Brassicaceae species-specific pathways using IAOX (indole-3-acetaldoxime) and IAN (indole-3-acetonitrile) as intermediates (Mano and Nemoto, 2012). All the above pathways synthesize TRP (L-tryptophan) as a precursor (Mano and Nemoto, 2012). Importantly, Glc increases the concentrations of many IAA precursors related to IAM, IAOX, and IAN pathways, as well as three major IAA metabolites and conjugates (oxIAA, IAAsp, and IAGlu), demonstrating a direct positive regulation of Glc in auxin synthesis (Sairanen et al., 2012). *In vivo* evidence consistent with this finding comes from *Arabidopsis* hypocotyls, where an endogenous carbon-sensing pathway triggers increased auxin flux and hypocotyl elongation (Lilley et al., 2012).Another important founding by Sairanen et al. (2012) is that the Glc-induced auxin synthesis is negatively regulated by the PHYTOCHROME-INTERACTING FACTOR (PIF) transcription factor family. Considering the up-regulation of PIF gene expression by Suc (Stewart et al., 2011), it is possible that PIF protein act as a switch-off button in Glc induction of auxin biosynthesis at high Suc level or by some specific developmental cues. Further evidence on the positive role of Glc in auxin biosynthesis comes from the maize *miniature1* mutant which lacks the expression of basal kernel-specific cw-Inv (INCW2), leading to miniature seed phenotype. In the mutant seed, the IPA auxin synthesis pathway was down-regulated through decreased expression of a maize *YUCCA* gene (LeClere et al., 2010). *YUCCA* encodes a flavin monooxygenase-like enzyme, which uses IPA as a substrate to produce IAA. This observation suggests that Inv-mediated generation of hexoses is required for auxin biosynthesis in developing seed.

In addition to its role in synthesis, Glc is also implicated in auxin distribution and signaling. To this end, microarray analysis revealed that Glc could regulate as much as 62% of IAA related genes in *Arabidopsis* seedlings, including those encoding auxin receptors TIR1 (transport inhibitor response 1) and ABP1, auxin transporter PIN1, auxin response factors ARF4, ARF8, and a number of genes belonging to auxin induced gene families such as AUX/IAA, GH3, and SAUR (Mishra et al., 2009). Studies using Glc-insensitive mutants also revealed that hexose-mediated sugar signaling partially functions through auxin response (Moore et al., 2003). Genetic studies showed that Suc and Glc stabilize N-MYC DOWN-REGULATED-LIKE1 (NDL1) protein, which interacts with Gβγ dimmers of heterotrimeric G-protein complex, thereby positively regulating auxin transport in root to promote lateral root initiation and emergence (Mudgil et al., 2009). During *Arabidopsis* embryogenesis, nuclear PI signaling was involved in regulating polar transport of auxin effluxer PIN1 via modulating endomembrane structure and trafficking (Luo et al., 2011). Given the importance of hexose substrate for PIs biosynthesis (see previous discussions), this observation implies a possible link of Glc-mediated auxin transport through PI signaling.

Apart from Glc, emerging evidence also indicates connections between the T6P/SnRK1 regulatory system and auxin signaling. For example, microarray analysis in *Arabidopsis* seedlings has demonstrated a down-regulation of *AUX/IAA* genes and auxin receptor gene *TIR1* by elevated T6P level (Paul et al., 2008). SnRK1 has been shown to interact with the SKP1 domain of the SCF complex and the 26S proteasome, possibly through phosphorylating targeted proteins such as AUX/IAA for degradation in SCF–TIR1 complex (Farras et al., 2001).

Most of the studies described above on sugar and auxin signaling were conducted in cell culture systems and vegetative tissues such as developing roots, hypocotyls, leaves, and young seedlings. Reproductive organs are equally, or even more complex than, vegetative tissues in response to sugar and hormonal signals (Ruan et al., 2012). We discussed this issue below by focusing on recent advance obtained from developing seed.

## ROLES OF SUGAR AND AUXIN SIGNALING IN SEED DEVELOPMENT

Angiosperm seeds originate from double fertilization, which give birth to the diploid embryo and the triploid endosperm, wrapped by the maternal tissue known as seed coat. Seed formation proceeds by a phase of cell division, which represents a crucial period for seed set, highly sensitive to biotic and abiotic stress, and impacting significantly on seed yield potential (Ruan et al., 2012).

During seed development, the endosperm mother cell initially undergoes nuclear divisions without cell wall formation to generate syncytial endosperm soon after fertilization (Olsen, 2004). Endosperm nuclear division occurs earlier and faster than the embryo cell proliferation (Bate et al., 2004; Nowack et al., 2007). This sequential endosperm and embryo proliferation are tightly coupled to different regulatory mechanisms, for example, sugar signaling. Recent findings on the asymmetric spatial expression of cw-Inv gene *GhCWIN1* in cotton embryo sac, implied a control of the sequential development of endosperm and embryo by Inv-mediated sugar signaling. This may be achieved through establishing a spatial gradient of Glc concentration being higher in the endosperm region than that in the embryo region, thus favoring endosperm nuclear division over embryo cell proliferation during the seed set phase (Wang and Ruan, 2012). Consistently, a cw-Inv inhibitor was localized at the boundary between the endosperm and embryo in developing maize seed (Bate et al., 2004), which could help to minimize hexose flow to young embryo, to ensure nuclear division in endosperm but a quiescent status in embryo at this stage.

Later, embryo develops starting from the acquisition of zygote polarity and elongation, follows by a serial of cell division. Soon after the asymmetric zygote division, the separated cells quickly establish an apical–basal axis of polarity, then the differentiation of an epidermis and the formation of the shoot and root meristem during the next rounds of cell division (Dumas and Rogowsky, 2008). Auxin plays a prominent role in regulating these pattern formations in a cell type-dependent manner (Rademacher et al., 2012). The molecular mechanisms of auxin action in early embryogenesis have been reviewed (e.g., Möller and Weijers, 2009; Lau et al., 2012), and will not be discussed here. Comparing to auxin, it remains unknown whether sugar signaling affects embryo pattern formation. However, significant correlations between Glc concentration and CWIN expression and early embryo (pre-heart stage) mitotic activity have been revealed in many species, such as *Arabidopsis*, faba bean, and cotton (Weber et al., 1996; Morley-Smith et al., 2008; Wang and Ruan, 2012), indicating Glc as a signal to stimulate cytokinesis during embryogenesis. Apart from Glc, T6P signaling may also affect mitosis activity in early embryo, as *Arabidopsis* *TPS1*-deficient mutant (*tps1*) shown slower embryo development compare to that of wild-type (Gómez et al., 2010). However, *tps1* embryos are eventually stopped at torpedo stage, implying T6P signal may not essential for rapid cell division in early development, but indispensable for the transition into late stage of embryo development (Gómez et al., 2010).

As embryo develops to heart-torpedo stages, cells undergo rapid expansion, and gradually start to accumulate storage materials. As discussed before, cell expansion could be regulated by the concerted actions of sugar and auxin signaling network. Many studies have shown the impacts of sugar and auxin on seed size (e.g., Cheng and Chourey, 1999; Andersen et al., 2002; Schruff et al., 2006). However, most of the observations are not able to differentiate signaling roles of sugar from its potential nutrient or osmotic effect. The contribution of auxin signal to seed size is largely due to its roles in cell division but not expansion. There is little direct evidence about the role of sugar and auxin signaling in cell enlargement so far, probably due to, in part, the complexity of cell types in seed.

By contrast, many studies have demonstrated that the switch from seed expansion to storage phase is strongly affected by sugar signals. A general finding is that the transition from cell division and expansion to storage activities in seed is usually associated with a decrease in Inv expression and activity and an increase in Sus activity, and an increased ratio of Suc to Glc. Using high-resolution histographical mapping, a clear correlation was observed between decreased Glc concentration and reduced mitotic activity in legume embryo (Borisjuk et al., 1998), largely due to the decrease of cw-Inv genes expression (Weber et al., 1995). In cytoplasm, an increase of Sus activity leads a shift in carbon use toward starch synthesis in plastid. For example, highest expression of *Sus* gene temporally matches with rapid starch filling in rice grain (Wang et al., 1999). In correlation to Suc level, T6P synthesis could be induced, which may then result in an increased expression and activity of the ADP-glucose pyrophosphorylase (AGPase), as well as the redox activation of AGPase, essential for starch accumulation in the plastid (O’Hara et al., 2013).

Clearly, much has been learned about the complexity of sugar and auxin network in regulating plant cell division and expansion. Further research on dissecting the complex network of sugar and auxin signaling pathways in specific plant tissues under particular growth stages or environment conditions, will not only contribute to a better understanding of the regulatory systems underpinning complex developmental processes, but could also have great importance in crop improvement through designing innovative approaches to optimize plant performance for yield and stress tolerance.

## Conflict of Interest Statement

The authors declare that the research was conducted in the absence of any commercial or financial relationships that could be construed as a potential conflict of interest.
